# Inhibition of Mcl-1 enhances cell death induced by the Bcl-2-selective inhibitor ABT-199 in acute myeloid leukemia cells

**DOI:** 10.1038/sigtrans.2017.12

**Published:** 2017-04-07

**Authors:** Daniel A Luedtke, Xiaojia Niu, Yihang Pan, Jianyun Zhao, Shuang Liu, Holly Edwards, Kang Chen, Hai Lin, Jeffrey W Taub, Yubin Ge

**Affiliations:** 1 Cancer Biology Graduate Program, Wayne State University School of Medicine, Detroit, Michigan, USA; 2 National Engineering Laboratory for AIDS Vaccine, School of Life Sciences, Jilin University, Changchun, China; 3 Department of Pathology and Laboratory Medicine, Weill Cornell Medicine, New York, New York, USA; 4 Department of Pediatrics, Wayne State University School of Medicine, Detroit, Michigan, USA; 5 Department of Oncology, Wayne State University School of Medicine, Detroit, Michigan, USA; 6 Molecular Therapeutics Program, Karmanos Cancer Institute, Wayne State University School of Medicine, Detroit, Michigan, USA; 7 Department of Obstetrics and Gynecology, Wayne State University School of Medicine, Detroit, Michigan, USA; 8 Mucosal Immunology Studies Team, National Institute of Allergy and Infectious Diseases, National Institutes of Health, Bethesda, Maryland, USA; 9 Department of Hematology and Oncology, The First Hospital of Jilin University, Changchun, China; 10 Division of Pediatric Hematology and Oncology, Children’s Hospital of Michigan, Detroit, Michigan, USA

## Abstract

Acute myeloid leukemia (AML) is a serious disease. The 5-year survival rates remain frustratingly low (65% for children and 26% for adults). Resistance to frontline chemotherapy (usually cytarabine) often develops; therefore a new treatment modality is needed. Bcl-2 family proteins play an important role in balancing cell survival and apoptosis. The antiapoptotic Bcl-2 family proteins have been found to be dysregulated in AML. ABT-199, a BH3 mimetic, was developed to target antiapoptotic protein Bcl-2. Although ABT-199 has demonstrated promising results, resistance occurs. Previous studies in AML show that ABT-199 alone decreases the association of proapoptotic protein Bim with Bcl-2, but this is compensated by increased association of Bim with prosurvival protein Mcl-1, stabilizing Mcl-1, resulting in resistance to ABT-199. In this study, we investigated the antileukemic activity of the Mcl-1-selective inhibitor A-1210477 in combination with ABT-199 in AML cells. We found that A-1210477 synergistically induced apoptosis with ABT-199 in AML cell lines and primary patient samples. The synergistic induction of apoptosis was decreased upon Bak, Bax and Bim knockdown. While A-1210477 treatment alone also increased Mcl-1 protein levels, combination with ABT-199 reduced binding of Bim to Mcl-1. Our results demonstrate that sequestration of Bim by Mcl-1, a mechanism of ABT-199 resistance, can be abrogated by combined treatment with the Mcl-1 inhibitor A-1201477.

## Introduction

Acute myeloid leukemia (AML) is a serious disease. In the United States, there are nearly twenty thousand new cases and over ten thousand deaths from this disease each year.^1^ The 5-year survival rates, 65% for children and 26% for adults, remain frustratingly low.^1^ While changes in treatment and survival outcomes have proven successful in other leukemia subtypes like pediatric acute lymphoblastic leukemia and chronic myeloid leukemia, little has changed in the treatment of AML over the course of the last four decades. Resistance often develops against the main drug of treatment, cytarabine, thus a new treatment modality is urgently needed for this deadly disease.

The Bcl-2 family of proteins plays a key role in balancing the decision between cell survival and apoptosis, and escape of apoptosis is a hallmark of cancer.^2–4^ This is especially important for the response to stress signals, including but not limited to cytotoxic agents like cytarabine and DNA damage. The Bcl-2 family has been found to be dysregulated in AML, with increases in the antiapoptotic family members Bcl-2, Bcl-xL and Mcl-1 having been found to play various roles.^5,6^ The BH3 mimetic ABT-199 was developed to target the antiapoptotic protein Bcl-2.^7,8^ ABT-199 improved upon its predecessor ABT-263 by not targeting Bcl-xL, whose inhibition leads to thrombocytopenia and limited clinical application.^7^ Results of a small phase I clinical trial have shown that ABT-199 has promising clinical activity, though a median time to relapse of 2.5 months indicates that resistance occurs quickly.^9,10^ Thus, ABT-199 may be most effective when used in combination therapies.

Prosurvival Bcl-2 family proteins, like Bcl-2, sequester Bim to prevent Bim from inducing apoptosis. Bim interacts with Bax/Bak leading to Bax/Bak activation. Activated Bax/Bak form pores in the mitochondria outer membrane, leading to release of cytochrome *c* and subsequent apoptosis. Targeting Bcl-2 with ABT-199 was expected to free Bim and induce apoptosis. Our previous studies in ABT-199-resistant AML cell lines and patients samples showed that ABT-199 decreased the association of Bim with Bcl-2 and increased the association of Bim with Mcl-1 (which along with Bcl-xL also acts as a prosurvival Bcl-2 family protein in a similar fashion to Bcl-2).^11^ In ABT-199-resistant cells, treatment with ABT-199 does not change the mitochondrial outer membrane permeabilization (MOMP), an event necessary for intrinsic apoptosis, pointing to changes in the balance of Bcl-2 family members as the primary reason for resistance.^11^ On the basis of this, it is evident that both Bcl-2 and Mcl-1 would have to be targeted to induce apoptosis in ABT-199-resistant AML cells.

Mcl-1 inhibition has evaded the current Bcl-2 inhibitors, but recent advances have yielded Mcl-1-specific inhibitors (such as A-1210477, a recently developed Mcl-1-specific inhibitor).^12^ Mcl-1 has been previously shown to decrease DNA damage and be necessary to inhibit Bak and Bax activation.^13–16^ The DNA damaging agent daunorubicin was found to decrease Mcl-1 levels and synergize with ABT-199.^11,17^ As Mcl-1 is upregulated in resistant cells, the induced DNA damage may counteract this mode of resistance. In this study, we found that ABT-199 and A-1210477 synergistically induce apoptosis in ABT-199-resistant and ABT-199-sensitive AML cells. ABT-199 and A-1210477 combination treatment disrupts binding of Bim with Bcl-2 and Mcl-1. This synergy was not dependent on DNA damage and occurred through the Bcl-2 family proteins.

## Materials and methods

### Drugs

ABT-199 and A-1210477 were purchased from Selleck Chemicals (Houston, TX, USA).

### Cell culture

THP-1 and U937 cell lines were purchased from the American Type Culture Collection (Manassas, VA, USA). MOLM-13 cells were purchased from AddexBio (San Diego, CA, USA). The cell lines have not been authenticated since receiving them in our laboratory. The cell lines were cultured in RPMI 1640 media with 10% fetal bovine serum (Life Technologies, Carlsbad, CA, USA) and 2 mM L-glutamine, plus 100 U ml^−l^ penicillin and 100 μg ml^−1^ streptomycin, in a 37 °C humidified atmosphere containing 5% CO_2_/95% air. Cell lines were tested for the presence of mycoplasma. Diagnostic AML blast samples derived from patients were purified by standard Ficoll-Hypaque density centrifugation, then cultured in RPMI 1640 with 20% fetal bovine serum, ITS Solution (Sigma-Aldrich, St Louis, MO, USA) and 20% supernatant of the 5637 bladder cancer cell line (as a source of granulocyte–macrophage colony-stimulating factor, granulocyte colony-stimulating factor, interleukin-1 beta, macrophage colony-stimulating factor and stem cell factor).^18–20^

### Clinical samples

Diagnostic AML blast samples were obtained from the First Hospital of Jilin University. Written informed consent was provided according to the Declaration of Helsinki. This study was approved by the Human Ethics Committee of The First Hospital of Jilin University. Clinical samples were screened for FLT3-ITD, NPM1, C-kit, CEBPA, IDH1, IDH2 and DNMT3A gene mutations and for fusion genes by real-time RT-PCR, as described previously.^18,21^ Patient characteristics are listed in Table 1. Normal peripheral mononuclear cells were derived from healthy donors.

### Western blot analysis

Cells were lysed in the presence of protease and phosphatase inhibitors (Roche Diagnostics, Indianapolis, IN, USA). Whole-cell lysates were subjected to SDS-polyacrylamide gel electrophoresis, electrophoretically transferred onto polyvinylidene difluoride (PVDF) membranes (Thermo Fisher Inc., Rockford, IL, USA) and immunoblotted with anti-Bcl-2 (ab692, Abcam, Cambridge, MA, USA), -Bcl-xL (2764), -Mcl-1 (4572), -PARP (9542), -Bim (2819), -γH2AX (2577), -Bak (3814), -Bax (2774), -cleaved caspase-3 (9661, designated -cf caspase-3, Cell Signaling Technology, Danvers, MA, USA) or -β-actin (A2228, Sigma-Aldrich) antibody, as previously described.^22,23^ Immunoreactive proteins were visualized using the Odyssey Infrared Imaging System (Li-Cor, Lincoln, NE, USA), as described by the manufacturer. Western blots were repeated at least three times and one representative blot is shown. Densitometry measurements were made using Odyssey V3.0 (Li-Cor), normalized to β-actin, and calculated as the fold-change compared to the corresponding no drug treatment control.

### Annexin V/PI staining and flow cytometry analysis

AML cells were treated with ABT-199 or A-1210477, alone or in combination, for 4 or 24 h and subjected to flow cytometry analysis using the annexin V-fluorescein isothiocyanate (FITC)/propidium iodide (PI) Apoptosis Kit (Beckman Coulter; Brea, CA, USA), as previously described.^24,25^ Results are expressed as percent annexin V+ cells. Experiments were performed three independent times in triplicate. For the AML cell lines, data presented are from one representative experiment, while patient sample experiments were performed once in triplicate due to limited sample size. Patient samples were chosen based on availability of adequate sample for the assay. The extent and direction of antileukemic interaction was determined by calculating the combination index (CI) values using CompuSyn software (Combosyn Inc., Paramus, NJ, USA). CI<1, CI=1 and CI>1 indicate synergistic, additive and antagonistic effects, respectively.^24,26^

### Immunoprecipitation

AML cell lines were treated for 4 h and then the cells were lysed using 1% CHAPS, 5 mM MgCl_2_, 150 mM NaCl, 1 mM EDTA, 1 mM EGTA, 20 mM Tris and 0.05% Tween-20 in the presence of protease inhibitors. Immunoprecipitation of Bim and Mcl-1 was performed as previously described^27^ using 2 μg of anti-Bim (2819, Cell Signaling Technology) or anti-Mcl-1 (SC-819, Santa Cruz Biotechnology, Santa Cruz, CA, USA) antibody, 1 mg protein lysate, and Protein A agarose beads (Roche Diagnostics). Proteins were eluted using 50 mM glycine, pH 2.0, and then analyzed by Western blotting.

### shRNA knockdown

The pMD-VSV-G and delta 8.2 plasmids were gifts from Dr Dong at Tulane University. Bim and non-target control (NTC) shRNA lentiviral vectors were purchased from Aldrich. Lentivirus production and transduction were carried out as previously described.^28^ Briefly, TLA-HEK293T cells were transfected with pMD-VSV-G, delta 8.2, and lentiviral shRNA constructs using Lipofectamine and Plus reagents (Life Technologies) according to the manufacturer’s instructions. Virus containing culture medium was harvested 48 h post transfection. Cells were transduced overnight using 1 ml of virus supernatant and 4 μg of polybrene and then cultured for an additional 48 h prior to selection with puromycin.

### Alkaline comet assay

U937 cells were treated for 4 h with ABT-199 and/or A-1210477 and subjected to alkaline comet assay as previously described.^28^ Slides were stained with SYBR Gold (Life Technologies), and then imaged on an Olympus BX-40 microscope equipped with a DP72 microscope camera and Olympus cellSens Dimension software (Olympus America Inc., Center Valley, PA, USA). Approximately 50 comets per gel were scored using CometScore (TriTek Corp, Sumerduck, VA, USA).

### Statistical analysis

Differences were compared using the two-sample *t*-test. Statistical analyses were performed with GraphPad Prism 5.0. Error bars represent±s.e.m. The level of significance was set at *P*<0.05.

## Results

### A-1210477 synergizes with ABT-199 in ABT-199-resistant AML cells

**Figure 1:**
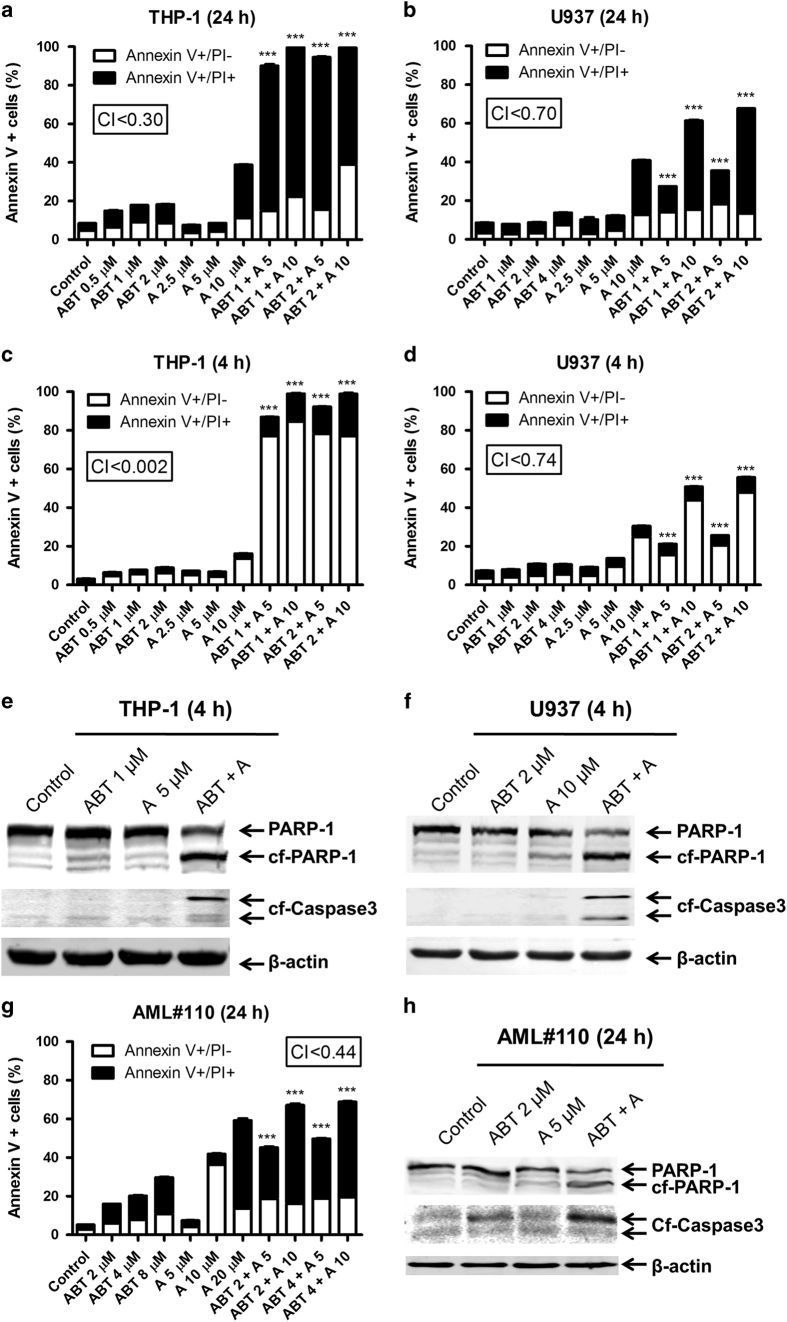
A-1210477 synergizes with ABT-199 to induce apoptosis in ABT-199-resistant acute myeloid leukemia (AML) cells. (**a**–**d**) THP-1 and U937 cells were treated with ABT-199 and A-1210477, alone or in combination, for 4 or 24 h and then subjected to annexin V/PI staining and flow cytometry analyses. ***P<0.001. Combination index (CI) values were calculated using CompuSyn software. (**e**, **f**) THP-1 and U937 cells were treated with ABT-199 or A-1210477 alone or in combination for 4 h. Whole-cell lysates were subjected to Western blotting and probed with the indicated antibodies. (**g**, **h**) Primary AML patient sample AML#110 was treated with ABT-199 and A-1210477, alone or in combination, for 24 h and then subjected to annexin V/PI staining and flow cytometry analyses (**g**). Whole-cell lysates were subjected to Western blotting and probed with the indicated antibodies (**h**). ***P<0.001. CI values were calculated using CompuSyn software.

To test our hypothesis that A-1210477 (abbreviated A) can synergize with ABT-199 (abbreviated ABT) to induce apoptosis, we tested various concentrations of ABT-199 and A-1210477 alone and in combination in ABT-199-resistant (U937, THP-1, and a primary AML patient sample derived at relapse) AML cells. The combination index (CI) was used to determine synergy. CI=1 denotes an additive effect while CI<0.9 denotes synergy, and CI<0.3 denotes strong synergy.^26^ At 24 h, synergy was observed between the two drugs for THP-1 (CI<0.30) and U937 (CI<0.70) cell lines (Figures 1a and b). Annexin V positive cells were largely propidium iodide (PI) positive as well, indicating that the cells were late apoptotic or necrotic.^29^ Annexin V/PI positivity was assessed after 4 h treatment to determine if cells may have undergone apoptosis. At 4 h, synergy was still observed for THP-1 (CI<0.002) and U937 (CI<0.74) cell lines and a majority of the Annexin V positive cells were PI negative, indicating that the cells underwent apoptosis (Figures 1c and d). Corroborating this, cleavage of PARP and caspase-3 was strongly enhanced in the combination treatment when compared to ABT-199 or A-1210477 alone in THP-1 and U937 cells (Figures 1e and f). Similar results were achieved in a primary patient sample *ex vivo* (Figures 1g and h). In summary, A-1210477 is able to synergize with ABT-199 to induce apoptosis in otherwise ABT-199-resistant AML cells in a synergistic manner.

### A-1210477 treatment increases Mcl-1 protein levels but releases Bim from Mcl-1

**Figure 2:**
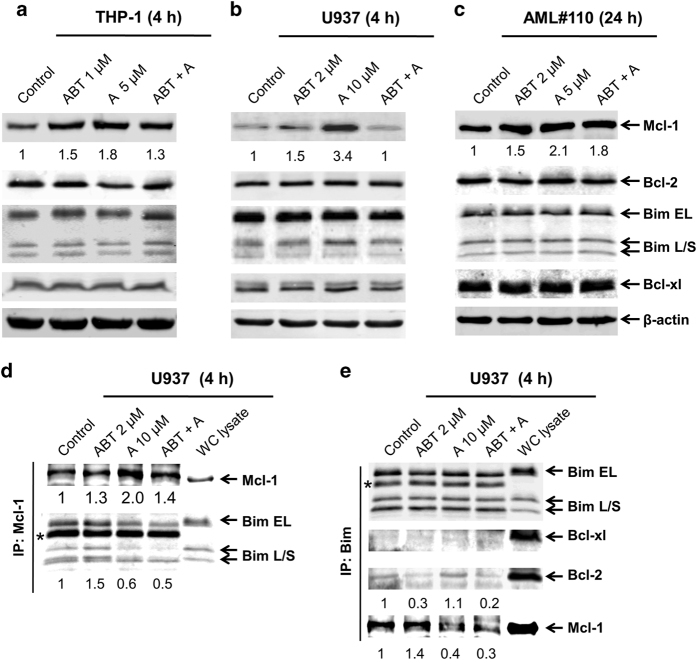
A-1210477 treatment increases Mcl-1 protein levels but releases Bim from Mcl-1. (**a**–**c**) THP-1, U937, and primary acute myeloid leukemia (AML) patient sample cells (AML#110) were treated with ABT-199 and A-1210477, alone or in combination, for 4 h or 24 h. Whole-cell lysates were subjected to western blotting and probed with the indicated antibodies. Relative densitometry measurements of Mcl-1 expression were measured using Odyssey Software V3.0. (**d**, **e**) U937 cells were treated with ABT-199 and A-1210477, alone or in combination, for 4 h. Mcl-1 (**d**) or Bim (**e**) was immunoprecipitated from whole-cell lysates and then subjected to western blotting and probed with the indicated antibodies. Relative densitometry measurements of Mcl-1, Bim and Bcl-2 were measured using Odyssey Software V3.0. ‘*’ indicates the light chain of the anti-Mcl-1 or -Bim antibody.

Having observed the synergy of ABT-199 and A-1210477, the next question was to determine how treatment affected levels of relevant Bcl-2 proteins and their interactions. Protein levels of Bcl-2, Mcl-1, Bcl-xL, and Bim were determined by Western blotting. Individual treatment with ABT-199 and A-1210477 caused increased levels of Mcl-1 without changing the levels of Bcl-2, Bim, and Bcl-xL in THP-1, U937, and a primary AML patient sample derived at relapse (Figures 2a–c). Combination treatment appeared to decrease Mcl-1 levels compared to A-1210477 alone, though levels remained similar to or higher than vehicle control treated cells, suggesting that disruption of the interaction of Mcl-1 with Bcl-2 family proteins played a critical role in the synergistic effects. To determine if interactions of Bcl-2 family members with Bim were disrupted by drug treatments, co-immunoprecipitation was performed. A-1210477 disrupted the interaction between Bim and Mcl-1 (Figure 2d), which surprisingly occurred despite the presence of elevated Mcl-1. The binding of Bim with Bcl-2 was disrupted by ABT-199 and not A-1210477 (Figure 2e). The binding of Bim with Bcl-2 and Mcl-1 was disrupted by the combination treatment. Bcl-xL, another antiapoptotic Bcl-2 family member which binds to Bim, did not compensate for this disruption, indicating that Bim was possibly unbound allowing for it to carry out its proapoptotic role.

### The effect of Bim, Bax and Bak knockdown on apoptosis induced by ABT-199 and A-1210477 in AML cells

**Figure 3:**
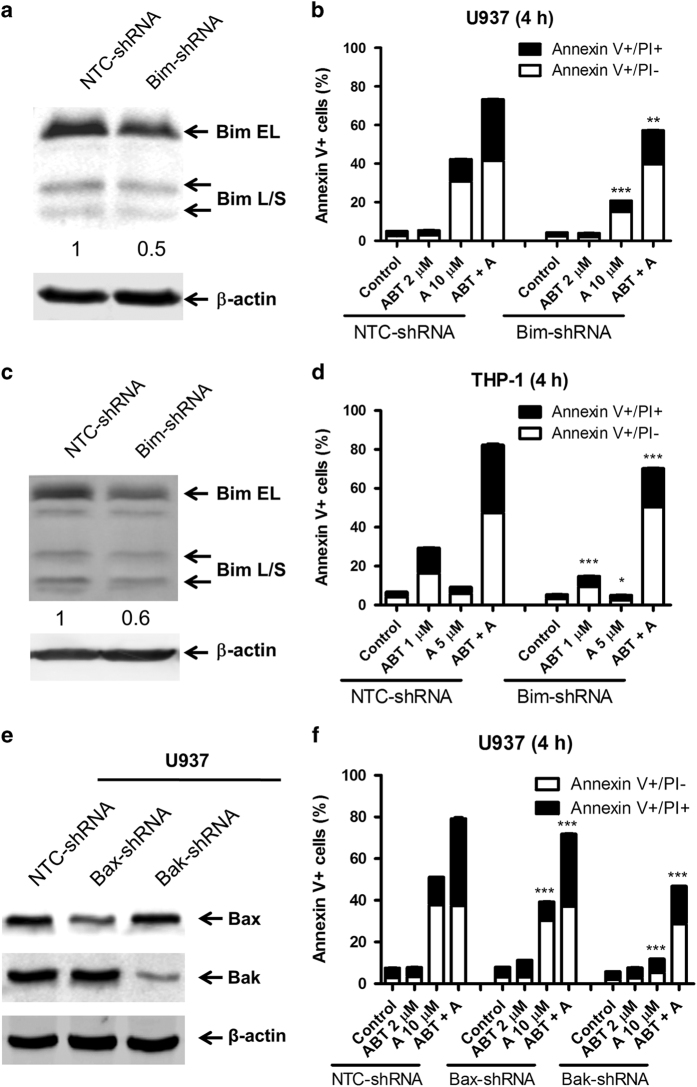
The effect of Bim, Bax and Bak knockdown on apoptosis induced by ABT-199 and A-1210477 in acute myeloid leukemia (AML) cell lines. (**a**, **c**) U937 (**a**) or THP-1 (**c**) cells were infected with non-template control (NTC-shRNA) or Bim (Bim-shRNA) shRNA lentivirus. Whole-cell lysates were subjected to western blotting and probed with the indicated antibodies to confirm the knockdown. (**b**, **d**) U937 (**b**) or THP-1 (**d**) shRNA knockdown cells were treated with ABT-199 and A-1210477, alone or in combination, for 4 h and then subjected to annexin V/PI staining and flow cytometry analyses. *P<0.05, **P<0.01 and ***P<0.001. (**e**) U937 cells were infected with non-template control (NTC-shRNA), Bax (Bax-shRNA) or Bak (Bak-shRNA) shRNA lentivirus. Whole-cell lysates were subjected to western blotting and probed with the indicated antibodies to confirm the knockdowns. (**f**) The U937 Bax and Bak knockdown cells were treated with ABT-199 and A-1210477, alone or in combination, for 4 h and then subjected to annexin V/PI staining and flow cytometry analyses. ***P<0.001.

To confirm the contribution of Bim to apoptosis induced by ABT-199 and A-1210477 combined treatment, shRNA knockdown was performed. Knockdown of Bim in U937 and THP-1 (Figures 3a and c) significantly reduced apoptosis in response to A-1210477 and combined treatment in both cell lines (Figures 3b and d). Bim knockdown also significantly reduced apoptosis induced by ABT-199 in THP-1 cells (Figure 3d). Bak and Bax knockdowns were performed in U937 cells due to the critical roles they play in the intrinsic apoptotic pathway (Figure 3e). As expected, knockdown of Bax or Bak (especially Bak) also reduced apoptosis significantly in the A-1210477 and combined treatments (Figure 3f). These results demonstrate that the canonical pathway of intrinsic apoptosis using Bim, Bak and Bax plays an important role in induction of apoptosis in response to A-1210477 treatment alone or in combination with ABT-199.

### A-1210477 induces DNA damage in AML cells

**Figure 4:**
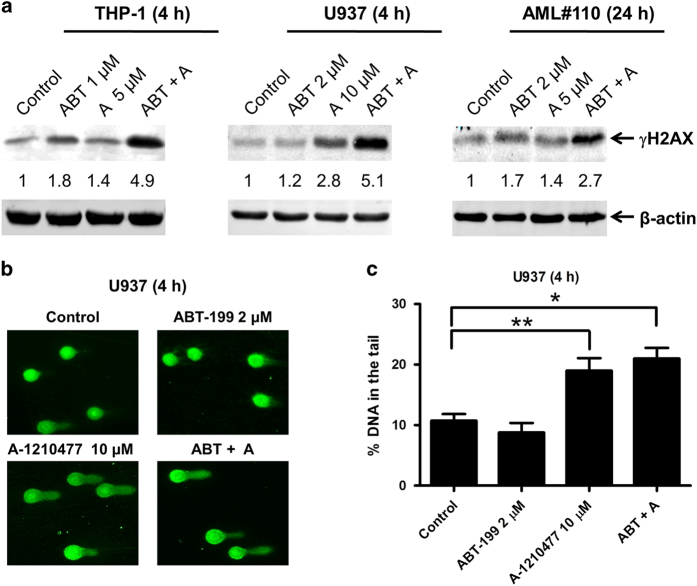
A-1210477 induces DNA damage in acute myeloid leukemia (AML) cells. (**a**) THP-1, U937, and a primary AML patient sample cells (AML#110) were treated with ABT-199 and A-1210477, alone or in combination, for 4 h or 24 h. Whole-cell lysates were subjected to Western blotting and probed with the indicated antibody. Relative densitometry measurements of γH2AX expression were measured using Odyssey Software V3.0. (**b**) U937 cells were treated with ABT-199 and A-1210477, alone or in combination, for 4 h and then subjected to alkaline comet analyses. Representative images are shown. (**c**) Alkaline comet assay results are graphed as median percent DNA in the tail from four replicate gels±s.e.m. *P<0.05 and **P<0.01.

Mcl-1 has a known role in both apoptosis and the DNA damage response. We previously demonstrated that ABT-199 treatment enhances DNA damage induced by DNA damaging agents,^11,30^ thus the question remained whether the synergistic action of A-1210477 combined with ABT-199 was due to apoptosis and/or DNA damage. γ-H2AX, a surrogate marker for DNA damage was measured by western blot.^31^ The level of γ-H2AX increased following ABT-199 or A-1210477 treatment, and increased after combined treatment in THP-1, U937, and a patient sample (Figure 4a). However γ-H2AX levels can indicate late apoptosis as well. To further elucidate the extent of DNA damage, the comet assay was performed. DNA damage, as measured by alkaline comet assay (Figures 4b and c), increased dramatically with A-1210477 treatment but not ABT-199 treatment. Interestingly, combination treatment did not increase DNA damage compared to A-1210477 treatment. While A-1210477 may induce some apoptosis by DNA damage, its synergistic interaction with ABT-199 is not due to DNA damage and is likely due to the apoptotic function of Mcl-1.

### A-1210477 synergizes with ABT-199 in ABT-199-sensitive AML cells

**Figure 5:**
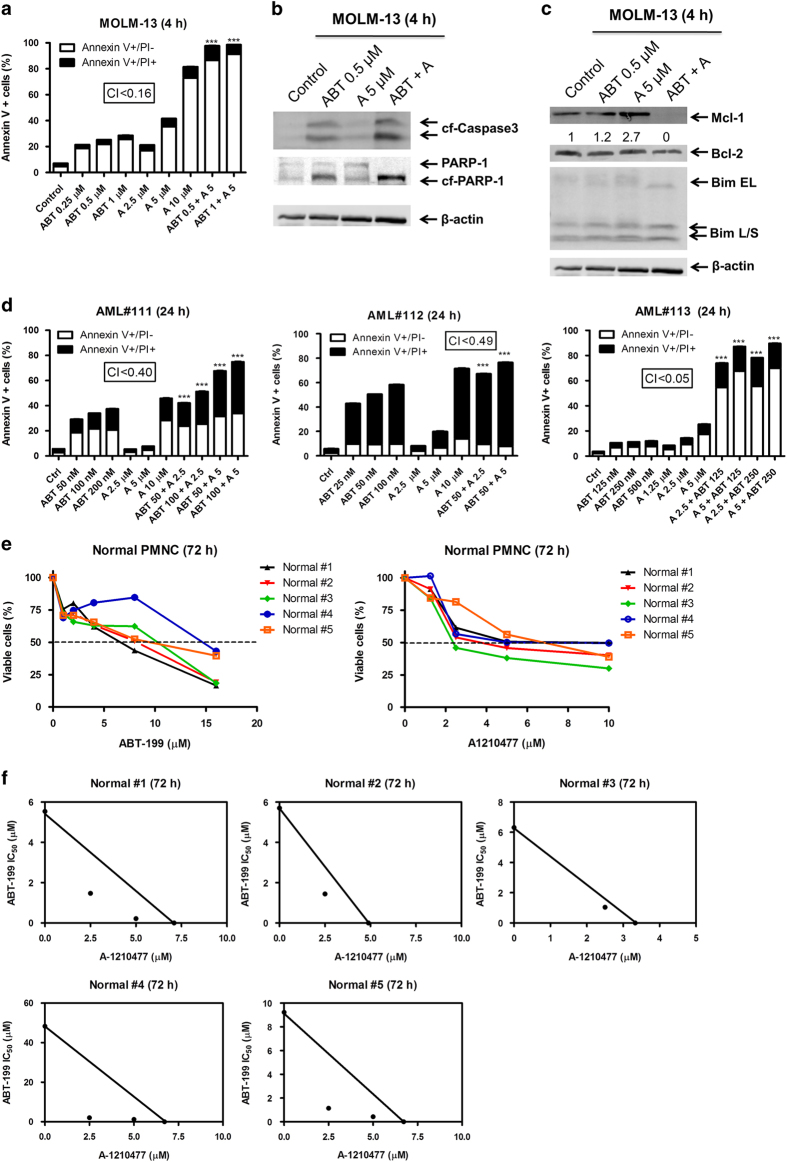
A-1210477 synergizes with ABT-199 in ABT-199-sensitive acute myeloid leukemia (AML) cells. (**a**) MOLM-13 cells were treated with ABT-199 and A-1210477, alone or in combination, for 4 h and then subjected to annexin V/PI staining and flow cytometry analyses. ***P<0.001. Combination index (CI) values were calculated using CompuSyn software. (**b**, **c**) MOLM-13 cells were treated with ABT-199 and A-1210477, alone or in combination, for 4 h. Whole-cell lysates were subjected to western blotting and probed with the indicated antibodies. (**d**) Primary AML patient sample cells (AML#111–113) were treated with ABT-199 and A-1210477, alone or in combination, for 24 h and then subjected to annexin V/PI staining and flow cytometry analyses. ***P<0.001. CI values were calculated using CompuSyn software. (**e**, **f**) MTT analyses were performed on normal peripheral blood mononuclear cells (PMNCs) to determine proliferation inhibition at the indicated concentrations of A-1210477 and ABT-199, alone or in combination. Patient sample data are means of duplicates due to limited sample. Standard isobologram analyses of drug interactions were performed to determine the extent and direction of the drug interactions. The IC_50_ values of each drug are plotted on the axes; the solid line represents the additive effect, while the points represent the concentrations of each drug resulting in 50% inhibition of proliferation. Points falling below the line indicate synergism whereas those above the line indicate antagonism.

To further increase the clinical relevance of the study, A-1210477 and ABT-199 combination treatment was performed in ABT-199-sensitive MOLM-13 cells, newly diagnosed AML patient samples, and normal donor peripheral blood mononuclear cells (PMNCs). Treatment of MOLM-13 with ABT-199 and A-1210477 synergistically induced apoptosis (Figure 5a, CI<0.16), accompanied by caspase-3 and PARP cleavage (Figure 5b). Similar to resistant cell lines, Bcl-2 and Bim levels were unchanged and Mcl-1 was induced by A-1210477 treatment alone (Figure 5c). However, Mcl-1 was not detected after combined treatment. Treatment of 3 newly diagnosed AML patient samples with A-1210477 and ABT-199 synergistically induced apoptosis, as well (CI<0.40, CI<0.49, CI<0.05, respectively, Figure 5d). In several relatively ABT-199-sensitive and -resistant AML cell lines and patient samples, ABT-199 and A-1210477 combination treatment synergistically induced apoptosis, suggesting that this combination may work regardless of ABT-199 sensitivity.

Lastly, to test the effects of combined treatment on normal cells in addition to AML cells, 5 normal donor peripheral blood mononuclear cells were subjected to single drug treatment of either ABT-199 or A-1210477 to determine IC_50_ values. ABT-199 IC_50_ values ranged from 5.5 to 48.3 μM, while A-1210477 IC_50_ values ranged from 3.3 to 7.1 μM (Figure 5e). IC_50_ values were also determined for A-1210477 in the presence of set concentrations of ABT-199. Surprisingly, it was found that A-1210477 and ABT-199 act synergistically to inhibit cell proliferation in normal PMNCs (Figure 5f).

## Discussion

It is widely believed that leukemic stem cells which have escaped chemotherapy treatment contribute to AML relapse.^32^ Although it has been shown that leukemic stem cells overexpress Bcl-2^33^ and that the Bcl-2-selective inhibitor ABT-199 has shown promise, leading to clinical trials in AML,^34^ resistance remains an issue. A phase I clinical trial showed a 19% complete response rate for ABT-199 monotherapy.^9^ However, all of the patients relapsed within a short period of time (median time to relapse was only 2.5 months), suggesting that ABT-199 would best be used in combination regimens.^9,10^ In this study, we demonstrated that inhibition of Mcl-1 enhanced ABT-199-induced apoptosis in both ABT-199-sensitive and ABT-199-resistant AML cells.

**Figure 6:**
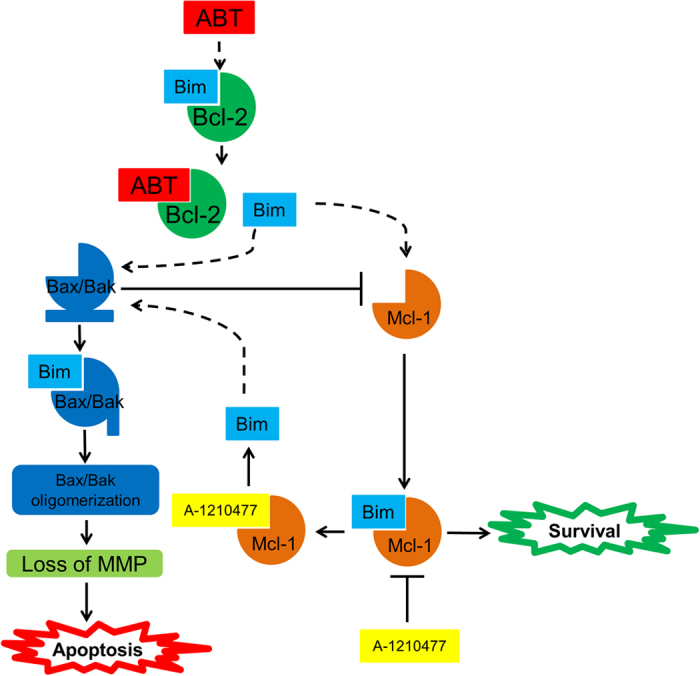
Proposed mechanism for ABT-199 in combination with A-1210477 in AML cells. ABT-199 treatment releases Bim from Bcl-2. In ABT-199-sensitive cells, there is an inadequate amount of Mcl-1 to sequester all of the released Bim, resulting in free Bim, which can then activate Bak/Bax, leading to apoptosis. In ABT-199-resistant cells, the Bim released from Bcl-2 is sequestered by Mcl-1, leading to stabilization of Mcl-1, and ultimately resulting in survival. In the combined drug treatment, addition of A-1210477 abolishes sequestration of Bim by Mcl-1, allowing Bim to activate Bax/Bak, resulting in enhanced apoptosis.

Previously, we reported that the Chk1 inhibitor LY2603618 induces DNA damage, decreases Mcl-1 levels (even in the presence of ABT-199), and synergistically induces apoptosis with ABT-199.^30^ In addition, we have also shown that DNA damage induced by chemotherapeutic agents is enhanced by ABT-199.^11,17^ While A-1210477 did cause DNA damage, enhanced DNA damage was not observed in the combined treatment, suggesting that DNA damage may not have played a role in the synergistic induction of apoptosis by the two agents in AML cells. On the basis of our current and previous findings, we propose the following mechanism of action of ABT-199 in combination with A-1210477. ABT-199 treatment releases Bim from Bcl-2. In sensitive cells, it is possible that there is not enough Mcl-1 to sequester all of the released Bim, resulting in free Bim, which can then activate Bax/Bak leading to apoptosis. In contrast, the Bim released from Bcl-2 is sequestered by Mcl-1, stabilizing Mcl-1 and ultimately resulting in survival in the ABT-199-resistant cells.^11^ Addition of A-1210477 reduces Mcl-1 interaction with Bim, decreasing sequestration of Bim, allowing for activation of Bax/Bak, and eventually resulting in apoptosis (Figure 6).

Although simultaneously inhibiting Bcl-2 and Mcl-1 results in synergistic induction of apoptosis in AML cells, the treatment also synergistically inhibited proliferation of normal PMNCs, indicating that toxicity may be a concern moving forward. While direct inhibition of Mcl-1 may be toxic, indirect targeting of Mcl-1 may prove to be more fruitful. We have previously demonstrated that DNA damaging agents daunorubicin, cytarabine, and CHK1-selective inhibitor LY2603618 downregulate Mcl-1 and enhance ABT-199-induced apoptosis in AML cells.^11,30^ It has been reported that dual PI3K/mTOR inhibitor, NVP-BEZ235, downregulates Mcl-1 and sensitizes leukemic cells to ABT-199.^35^ Inhibition of CDK9 has also been shown to enhance ABT-199 sensitivity through downregulation of Mcl-1.^36^ In summary, the Mcl-1 inhibitor A-1210477 enhances ABT-199-induced apoptosis in AML cells, though synergistic results were also revealed in normal PMNCs. These results warrant pursuing new Mcl-1 inhibitors or therapies, which indirectly inhibit Mcl-1 for combination with ABT-199 in AML.

## Figures and Tables

**Table 1:** Patient characteristics for primary AML patient samples

*Patient*	*Gender*	*Age (years)*	*Disease Status*	*FAB subtype*	*Cytogenetics*	*Blast purity (%)*	*Gene mutation*
AML#110	Female	66	Relapsed	M4	45, XX, −7	48.0	
AML#111	Male	58	Newly diagnosed	M2	46, XY	63.5	CEBPα double mutation, DNMT3A mutation, GATA2 mutation
AML#112	Male	46	Newly diagnosed	M1	46, XY	92.0	CEBPα double mutation
AML#113	Female	10	Newly diagnosed	M5	46, XX	97.0	

Abbreviation: AML, acute myeloid leukemia.
